# #toolittletoolate: JUUL-related content on Instagram before and after self-regulatory action

**DOI:** 10.1371/journal.pone.0233419

**Published:** 2020-05-21

**Authors:** Lauren Czaplicki, Shreya Tulsiani, Ganna Kostygina, Miao Feng, Yoonsang Kim, Siobhan N. Perks, Sherry Emery, Barbara Schillo

**Affiliations:** 1 Schroeder Institute at Truth Initiative, Washington, DC, United States of America; 2 Social Data Collaboratory, NORC at the University of Chicago, Chicago, IL, United States of America; Medical University of South Carolina, UNITED STATES

## Abstract

**Introduction:**

Digital e-cigarette marketing is largely unregulated and remains easily accessible to young people. The growing public concern around youth JUUL use and its viral presence on social media led the company to engage in several voluntary actions to remove and reduce JUUL-related content on Instagram in May 2018. The current study examined how JUUL-related Instagram content changed in the US following JUUL Labs’ wave of voluntary actions in May 2018.

**Methods:**

In 2019, we collected a total of 50,817 JUUL-relevant posts by 16,323 unique users on Instagram from March 1-May 15, 2018 (Phase 1) and May 16-November 11, 2018 (Phase II) using the application programming interface. We conducted a semantic network analysis to identify major topic clusters over time.

**Results:**

Approximately 14,838 JUUL-related posts were made by 5,201 accounts in Phase I and 35,979 posts were made by 11,122 accounts in Phase II. Major content clusters remained unchanged over time–key topics were JUUL-related product characteristics and JUUL-communities; the general vape community; and cannabis-related behavior. Of note, cannabis-related content grew in Phase II, particularly use of the term CBD.

**Conclusions:**

Our results reflect the limits of voluntary industry actions to reduce or change vaping-related content on social media. Rather, strong federal restriction on commercial tobacco marketing is the optimal pathway to reduce initial product marketing exposure among youth. These limits would make the emergence and viral contagion of brand-related social media content less likely and reduce its influence on youth behavior.

## Introduction

There are currently no federal laws banning digital tobacco or e-cigarette marketing. [1] Commercial e-cigarette content remains easily accessible to young people, [1, 2] and a growing body of research highlights the prevalence of such content on sites popular with youth, like Instagram. [3–7]

Since early 2018, the e-cigarette market in the US has been dominated by JUUL. [3, 8, 9] JUUL is a high-nicotine content device tied to the recent epidemic rise in e-cigarette use among youth in the US. [10] JUUL’s success was partly due to its early social media marketing campaign, “Vaporized” which was launched in 2015 and featured youthful models and pop-culture references. [8, 11] This campaign helped solidify JUUL’s youth-friendly brand image [8] and JUUL’s presence on social media virally grew to include content posted by other commercial, vape community, and individual user accounts. [3, 12] JUUL appeals to youth for many reasons (e.g. flavor, design), [13] and exposure to JUUL-related social media content may be one key factor in promoting and normalizing product use among youth. [3, 8]

To address growing federal and public concern over JUUL’s popularity among teens, [14] JUUL Labs took several actions to “remove posts, pages, and unauthorized offers to sell its products targeted at underage users” on social media, particularly Instagram. [15] In mid-May 2018, the company requested Instagram remove several popular unofficial community accounts (e.g. @juulcentral, @juulnation, @Doit4juul), many of which boasted hundreds of thousands of followers and some, like “Doit4juul,” which encouraged users to create their own JUUL-related content. [11, 15, 16] In July 2018, a spokesperson claimed that the company removed 4,000 youth-oriented JUUL-related posts across Instagram and Facebook. [17] JUUL Labs also deleted all corporate Instagram content posted prior to June 17, 2017, including those related to their “Vaporized” campaign in mid-2018 and shut down the official @juulvapor Instagram account on November 14, 2018. [8, 12]

Corporate self-regulatory actions such as those implemented by JUUL appear insufficient to deter product-related behavior, [18, 19] or reduce youth exposure to pro-vaping content. Preliminary evidence suggests that following the removal of @juulvapor, Instagram posts using #juul increased from 260,866 in late October 2018 to over 330,000 in January 2019. [8] In the current study, we expand this evidence base to document if and how JUUL-related content on Instagram changed following JUUL Labs’ voluntary actions in May 2018. Results can enhance understanding of how social media content may change following industry self-regulatory efforts and inform federal regulation.

## Methods

JUUL-related posts were collected from the Instagram application programming interface (API) through NUVI, Inc., a licensed syndicator of the Instagram data obtained from the platform’s API. Data were retrieved prospectively, in real time during the period of data collection and collected in accordance with the terms and conditions of the API. NUVI produced a collection of de-duplicated posts that contained at least one of search term in the body of the primary Instagram posts. In contrast to other APIs to collect social media (e.g. Twitter), NUVI does not match search terms against commentary or replies to the primary post. Therefore, our sample only included primary Instagram posts.

We used a previously documented set of 50 search terms to retrieve data (see Table 1). The search terms were hashtags related to “juul” and account handles, including @juulvapor and known handles and hashtags for JUUL-related community accounts, with the highest number of followers at the time of data collection based on prior research (e.g. @juulcentral, @juulnation, @juul.girlz). [3, 11] These community accounts were not identifiable with an individual person but referred to peer group behavior related to JUUL use/general vaping or promoted juuling/vaping as a lifestyle activity.

**Table 1:** Hashtag search terms used to retrieve JUUL-related posts from Instagram.

JUUL hashtag search terms
#doit4juul, #girlswhojuul, #justjuul, #juuhit, #juul, #juul_university, #juulbabes, #juulbabies, #juulbong, #juulboys, #juulbreak, #juulcentral, #juulchin, #juulcompatible, #juulers, #juulfanatics, #juulfavorite, #juulforsale, #juulforyou, #juulgang, #juulgirls, #juulgirlz, #juulhigh, #juulhighschool, #juulhumor, #juuling, #juulinstock, #juuljob, #juulkit, #juullife, #juullovers, #juulluge, #juullyfe, #juulmangopods, #juulmoment, #juulnation, #juulpen, #juulpod, #juulpods, #juulrealm, #juuls, #juulsjuuls, #juulskins, #juulstarterkit, #juulstick, #juulvapar, #juulvape, #juulvapes, #juulvapor, #switchtojuul

Data was collected in 2019 and occurred in two stages: Phase I (March 1-May 15, 2018) and Phase II (May 16-November 11, 2018). Data collection terminated in mid-November due to actions taken by Facebook, Instagram’s parent company, to eliminate third-party access to public Instagram posts by December 11, 2018.

### Data cleaning

Two coders rated a stratified random sample of 619 posts to determine if content was JUUL-related based on visual and language components. This labeled sample trained a linear support vector machine, or a supervised model with associated learning algorithms used for classifying text, [20] to remove irrelevant posts (recall and precision > 0.90). The final analytical dataset included 50,817 JUUL-relevant posts by 16,323 unique users.

### Statistical analysis

We used Wordij 3.0, NodeXL Pro, and Gephi to conduct a hierarchical semantic network analysis (SNA) of terms included in all primary JUUL-related Instagram posts. SNA is an inductive text-based approach to identify major clusters of interconnected terms based on the frequency with which terms appear and the number of co-occurrences between terms. [21] Although Instagram is largely image driven, there is strong evidence to suggest that tags used to describe photos largely correspond to image content. [22] Separate SNA’s were conducted for Phase I and Phase II. We created a network map based on the 100 most frequently occurring terms in each phase to identify major content clusters. Terms included both hashtags and text used in the post captions. Colors highlighted each major cluster and terms could appear in multiple clusters. Font size of the term increased or decreased based on its frequency of occurrence across posts and width of the lines between terms was thinner or thicker based on frequency of co-occurrence between the pair. Data from the two most active accounts posting multiple times a day (@thebombestheadshop, @vapesweaters) were excluded to reduce bias.

We used LOESS smoothing curve to visualize the trend of relevant posts over time. [23] We also conducted Joinpoint regression to examine changes in the temporal trend in the number of JUUL-related posts made per day from March 1-November 11, 2018. Using Joinpoint regression, we estimated average percentage change in number of posts per day and its corresponding 95% confidence interval (95% CI). [24]

## Results

**Fig 1:**
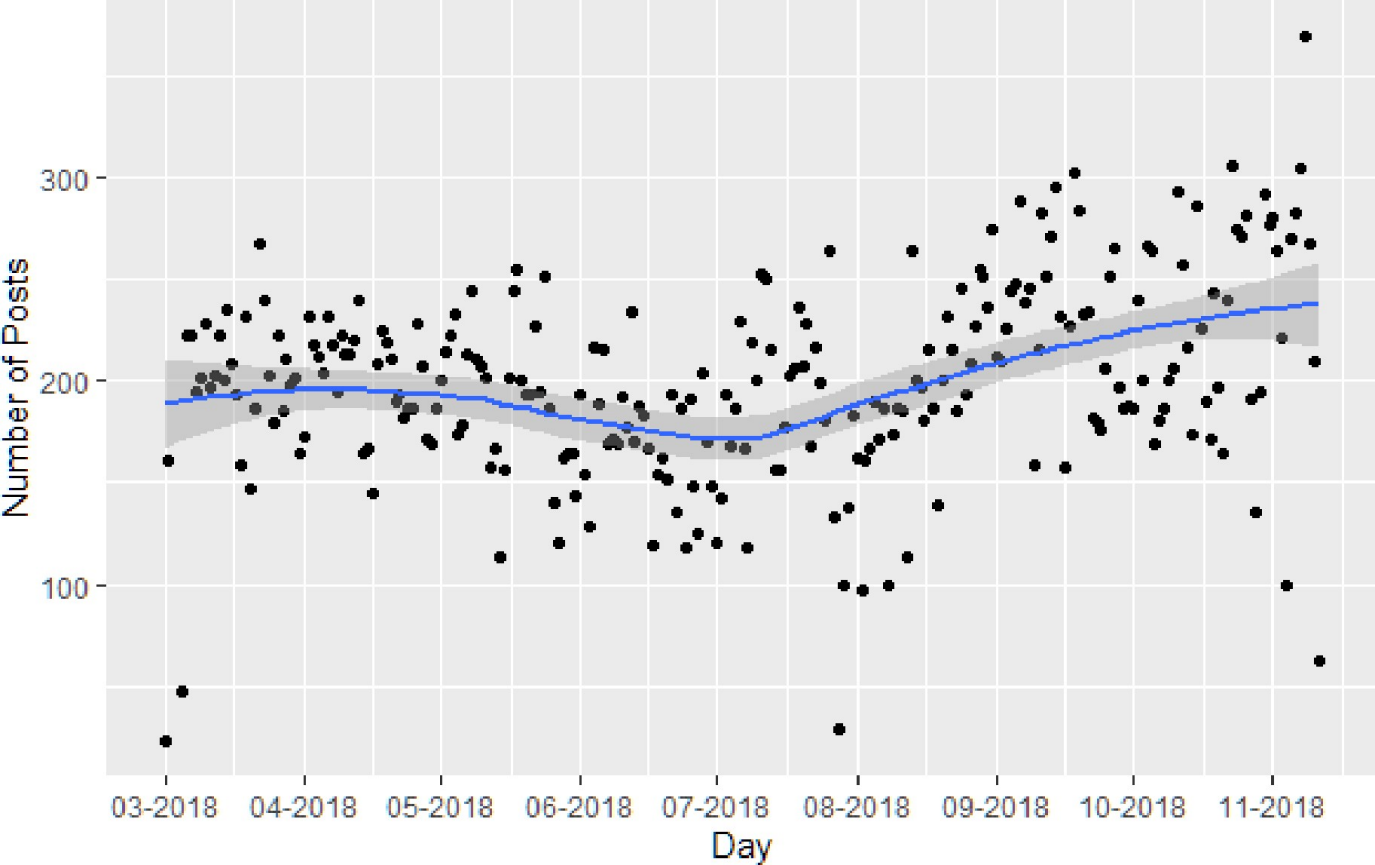
Trendline analysis of JUUL-related Instagram posts over time (March 1-November 11, 2018).

Our search captured 14,838 JUUL-related posts by 5,201 accounts in Phase I and 35,979 posts by 11,122 accounts in Phase II. Fig 1 depicts the trendline of daily number of posts during the data collection period and the LOESS curve. Based on Joinpoint regression, a significant change in trend was detected on June 24, 2018 (p = 0.001). The number of posts changed -0.2% (95% CI -0.3 to 0.0) per day from May 1 to June 24, and subsequently increased 0.3% (95% CI 0.2 to 0.4) per day from June 25 to November 11. The LOESS curve shows that the decline may have started from late May, but Joinpoint regression did not detect this change as significant.

**Fig 2:**
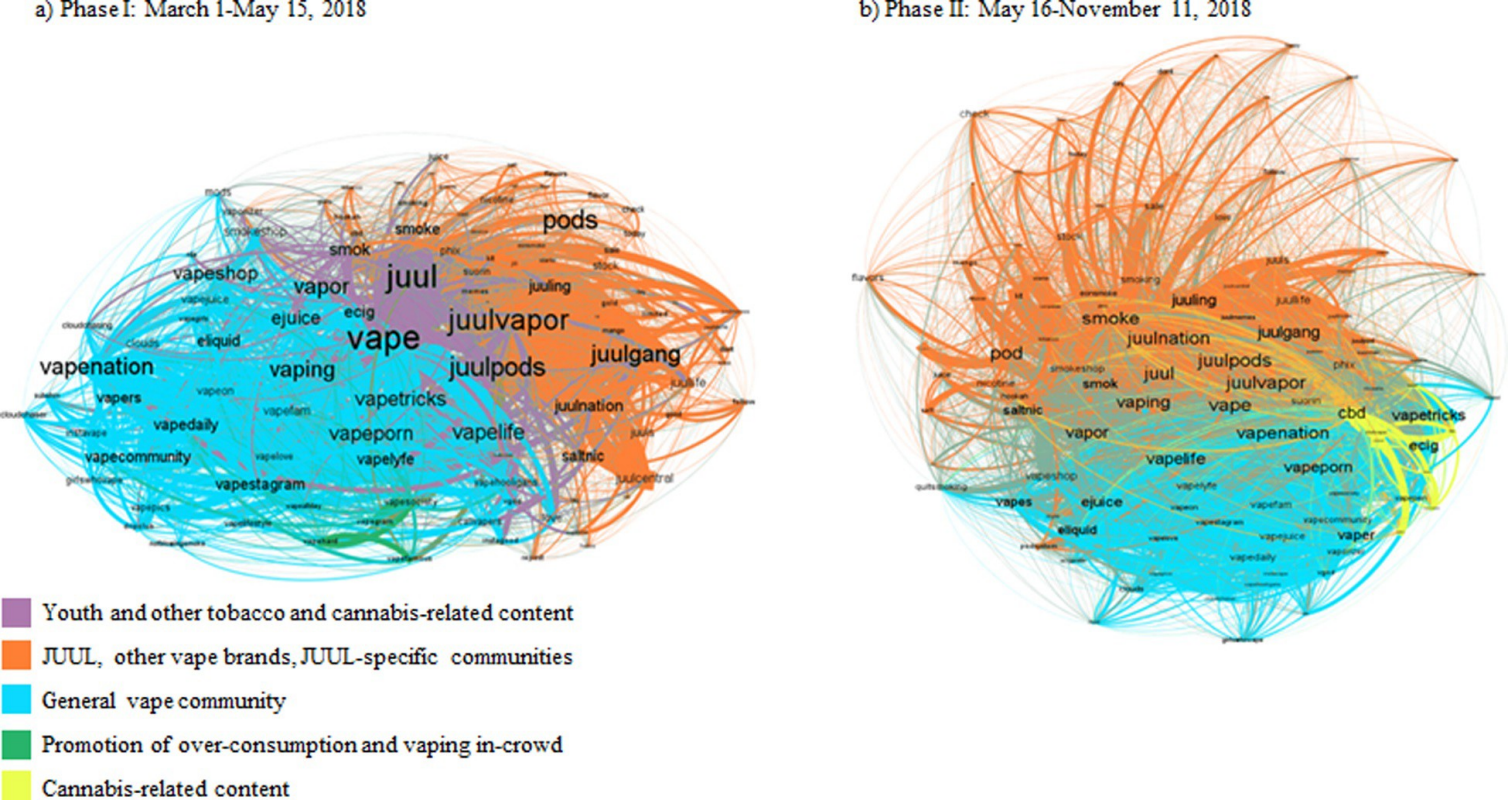
Network analysis of JUUL-related Instagram content (a) before and (b) following self-regulatory actions taken by JUUL Labs to limit social media presence. Colors are used to differentiate clusters of terms; the label font size corresponds to the frequency of occurrence of a term; edges/ties represent co-occurrence within three-term distance and width of the lines between terms is thinner or thicker based on the frequency of co-occurrence between the pair. Across Phases I and II, orange color corresponds to JUUL community-related content; blue color—vape community-related content.

Fig 2 graphically displays semantic network analyses results. Overall, the semantic structure of the clusters was similar between Phase I and Phase II. The terms “vape,” “juul,” “juulpods,” and “juulvapor” remained centrally placed in each network.

There were four major clusters in Phase I: Cluster a-I (purple lines) depicted youth and other tobacco (e.g., hookah) and cannabis-related content; Cluster a-II (orange) focused on JUUL product characteristics, JUUL-specific communities (e.g. juulgang), and other vape products/brands (e.g. Phix, EonSmoke); Cluster a-III (blue) focused on the general vape community (e.g. vapenation); and the smallest Cluster a-IV (green) represented content related to the promotion of over-consumption and vaping in-crowd (e.g. vapefamous, vapehard).

In Phase II, cannabis-related content (Cluster b-I, yellow lines) grew more than expected accounting for the fact that Phase II data collection was 2.8 times longer than Phase 1. The frequency of the term CBD increased from 562 occurrences in Phase I to 3,858 in Phase II and the number of links to other terms increased from 143 to 739. The other two major clusters in Phase II were the general vape community (Cluster b-II, blue) and JUUL product characteristics, JUUL-specific communities, and other vape products/brands (Cluster b-III, orange). Terms like smoke, smoking and hookah, which were more closely tied to cannabis-related content in Phase I become more central to the JUUL-specific community in Phase II. Additionally, content related to vaping in-crowd and overconsumption was more greatly tied and merged in the larger general vape community cluster in Phase II. The frequency at which other vape brands such as the JUUL-like device Phix and JUUL-compatible brand Eonsmoke were mentioned was similar in both phases.

## Discussion

We found that the volume of JUUL-related Instagram posts, as displayed in Fig 1, did not appreciably change following JUUL Labs’ first wave of content removal in May 2018. There was a significant, albeit slight, decline in the average number of daily posts May through late-June, which likely reflects Instagram and JUUL Labs’ actions to remove JUUL-related posts and active community accounts. [11, 15–17] However, the number of daily posts subsequently rebounded and grew from July through November. This net increase may be the artifact of many factors (e.g. natural growth in JUUL’s popularity; [25] re-generation of deleted community accounts posting under new names; [26] increase in news coverage around JUUL), but ultimately demonstrates that the self-regulatory actions undertaken did not decrease the overall volume of JUUL-related content.

With respect to the network analysis, we observed a notable increase in the prominence and frequency of cannabis-related terms in Phase II. This finding may reflect shifting patterns in cannabis and vape behavior over time, and foreshadows the growing concern over lung illness and vaping unofficial cannabis-derived constituents. [27] Nevertheless, results from our semantic network analyses demonstrate that major topics discussed remained relatively unchanged. These data suggest youth were likely exposed to the same JUUL-related Instagram content over time, regardless of JUUL Labs’ self-regulatory activities.

Digital marketing inherently leverages user-generated content to promote brands through peer-to-peer, electronic word-of-mouth. [28, 29] JUUL’s early social media campaigns are no exception and were successful in prompting a proliferation of JUUL-related content across commercial, community, and individual user accounts. One example is the aforementioned “Doit4juul” social media campaign, which was initiated by a commercial vendor of JUUL-compatible products. Organic users, or nonpaid consumers of social media platforms, were encouraged to post pictures or videos on their own Instagram page documenting their experience using JUUL. [11] While this campaign was not sanctioned by the official JUUL account, it demonstrates how the popularity of the product on social media was leveraged by others to reach organic users and their networks. The greatest acknowledgement of the proliferation of JUUL-related content was JUUL Labs’ own public and voluntary response to work with social media platforms to remove third-party unauthorized commercial and vape-community content, including “Doit4juul.” [12, 15]

Our results demonstrate the limits of this voluntary action and challenge whether industry-led efforts can effectively reduce pro-vaping social media content, particularly after an account and its posts have already developed a following. Instead, federal standards completely restricting digital commercial e-cigarette content are necessary to prevent another product like JUUL from utilizing the power of social media to target youth and young adults. Other countries, like the United Kingdom, have adopted standards that restrict commercial tobacco advertising on social media platforms like Instagram. [30] Although there are inherent challenges in regulating commercial social media content given that posts can be shared across national borders, [31, 32] a global body of evidence suggests that comprehensive tobacco advertising restrictions across traditional and digital media outlets can reduce rates of youth initiation and exposure to environmental cues that support tobacco use. [32–36]

Importantly, restrictions on commercial vaping advertising on social media platforms would not limit individual speech. Rather, they would eliminate one of the essential components needed to create a viral social media marketing campaign–an effective brand message that users share within their networks and begin to discuss organically. [28, 29] Such measures make the emergence and viral contagion of brand-related social media content less likely and reduce its potential influence on youth behavior. These recommendations have implications beyond vaping and can apply to other industries (e.g. fast-food) that use corporate campaigns to encourage user-generated content that promotes product use (e.g. #HowDoYouKFC). [36]

### Limitations

The present study was limited to the search terms used and may not have captured the full universe of JUUL-related content on Instagram during the study time period. Additionally, for ease of interpretation we only conducted cluster analyses using the top 100 terms in each dataset. These were the most frequently occurring terms and integral to overall network structure; however, it is possible that less frequently occurring terms could uniquely cluster together. The methods used also did not account for post imagery. Thus, we are limited in our ability to examine the inter-relational structure of concepts within images and between images and text to create an even more robust network of topic clusters. Finally, we looked at content across all account types (e.g. commercial, community, organic user) to demonstrate global change in Instagram content over time. However, future studies with access to account level data should investigate how content changed over time by user type.

## Conclusions

Our study demonstrates the ineffectiveness of industry-led efforts to reduce exposure to e-cigarette-related social media content among youth and highlights the need for federal restrictions on commercial e-cigarette marketing. Strong regulation is required to reduce youth exposure to pro-vaping content online and reduce e-cigarette use among young people. Further, findings can inform the development of digital marketing restrictions to industries beyond vaping corporations like JUUL.
